# Persistence of immunity and impact of third dose of inactivated COVID-19 vaccine against emerging variants

**DOI:** 10.1038/s41598-022-16097-3

**Published:** 2022-07-14

**Authors:** Krishna Mohan Vadrevu, Brunda Ganneru, Siddharth Reddy, Harsh Jogdand, Dugyala Raju, Gajanan Sapkal, Pragya Yadav, Prabhakar Reddy, Savita Verma, Chandramani Singh, Sagar Vivek Redkar, Chandra Sekhar Gillurkar, Jitendra Singh Kushwaha, Satyajit Mohapatra, Amit Bhate, Sanjay Kumar Rai, Raches Ella, Priya Abraham, Sai Prasad, Krishna Ella

**Affiliations:** 1 Bharat Biotech International Limited, Genome Valley, Hyderabad, 500 078 India; 2 Indian Council of Medical Research-National Institute of Virology, Pune, India; 3 Nizam’s Institute of Medical Sciences, Hyderabad, India; 4 Pandit Bhagwat Dayal Sharma Post Graduate Institute of Medical Sciences, Rohtak, India; 5 All India Institute of Medical Sciences, Patna, India; 6 Redkar Hospital, Dargalim, India; 7 Gillurkar Hospital, Nagpur, India; 8 Prakhar Hospital, Kanpur, India; 9 SRM Hospital and Research Centre, Kattankulathur, India; 10 Jeevan Rekha Hospital, Belgaum, India; 11 All India Institute of Medical Sciences, New Delhi, India; 12 Independent Clinical Development Consultant, Cambridge, USA

**Keywords:** Infectious diseases, Biotechnology, Diseases, Medical research, Immunology, Vaccines

## Abstract

This is a comprehensive report on immunogenicity of COVAXIN^®^ booster dose against ancestral and Variants of Concern (VOCs) up to 12 months. It is well known that neutralizing antibodies induced by COVID-19 vaccines wane within 6 months of vaccination leading to questions on the effectiveness of two-dose vaccination against breakthrough infections. Therefore, we assessed the persistence of immunogenicity up to 6 months after a two or three-dose with BBV152 and the safety of a booster dose in an ongoing phase 2, double-blind, randomized controlled trial (ClinicalTrials.gov: NCT04471519). We report persistence of humoral and cell mediated immunity up to 12 months of vaccination, despite decline in the magnitude of antibody titers. Administration of a third dose of BBV152 increased neutralization titers against both homologous (D614G) and heterologous strains (Alpha, Beta, Delta, Delta Plus and Omicron) with a slight increase in B cell memory responses. Thus, seronversion rate remain high in boosted recipients compared to non-booster, even after 6 months, post third dose against variants. No serious adverse events observed, except pain at the injection site, itching and redness. Hence, these results indicate that a booster dose of BBV152 is safe and necessary to ensure persistent immunity to minimize breakthrough infections of COVID-19, due to newly emerging variants.

**Trial registration:** Registered with the Clinical Trials Registry (India) No. CTRI/2021/04/032942, dated 19/04/2021 and on Clinicaltrials.gov: NCT04471519.

## Introduction

The emergence of SARS-CoV-2 variants of concern (VOCs) has raised concerns about the breadth and durability of neutralizing antibody responses^1^. Diminished vaccine effectiveness against VOCs such as Alpha (B.1.1.7), Beta (B.1.351), Delta (B.1.617.2), and Omicron (B.1.1.529) has been reported for several authorised vaccines with two doses of vaccination^2–9^. With the potential of newly emergent highly transmissible VOCs, illustrated by the recent circulation of the Omicron variant^10^, understanding the persistence of neutralizing antibody responses against VOCs has become vital to assess the need for additional booster dose. BBV152 is a whole-virion inactivated SARS-CoV-2 vaccine formulated with a Toll-like receptor 7/8 agonist molecule adsorbed onto alum (Algel-IMDG). We previously reported interim findings from a phase 2 controlled, randomized, double-blind trial on the immunogenicity and safety of two different formulations of BBV152 intended to select one formulation for further clinical development^11^. Based on tolerable safety outcomes and humoral and cell-mediated responses, the 6 µg dose with Algel-IMDG was selected for assessment in a phase 3 efficacy trial in which we demonstrated an overall vaccine efficacy of 77.8% (95% CI 65.2–86.4) against any COVID-19 and 65.2% (95% CI 33.1–83.0) efficacy against the Delta (B.1.617.2)^12^.

Trial participants in the phase 2 trial (here after referred to as the parent study) were followed up until 6 months after the second dose to evaluate the durability of immune responses. Following a protocol amendment and obtention of new consent, we randomized participants who previously received the 6 µg dose with Algel-IMDG to receive either a third (booster) dose of BBV152 or placebo (here after referred as non-booster). Here, we report, findings on the immunogenicity and safety of third (booster) dose of BBV152 with a significant protective efficacy in booster recipients compared to non-booster recipients, upto 12 months from the primary vaccination series.

## Results

### Neutralization antibodies decline in magnitude but persist above the baseline at 6 months post second dose

**Figure 1:**
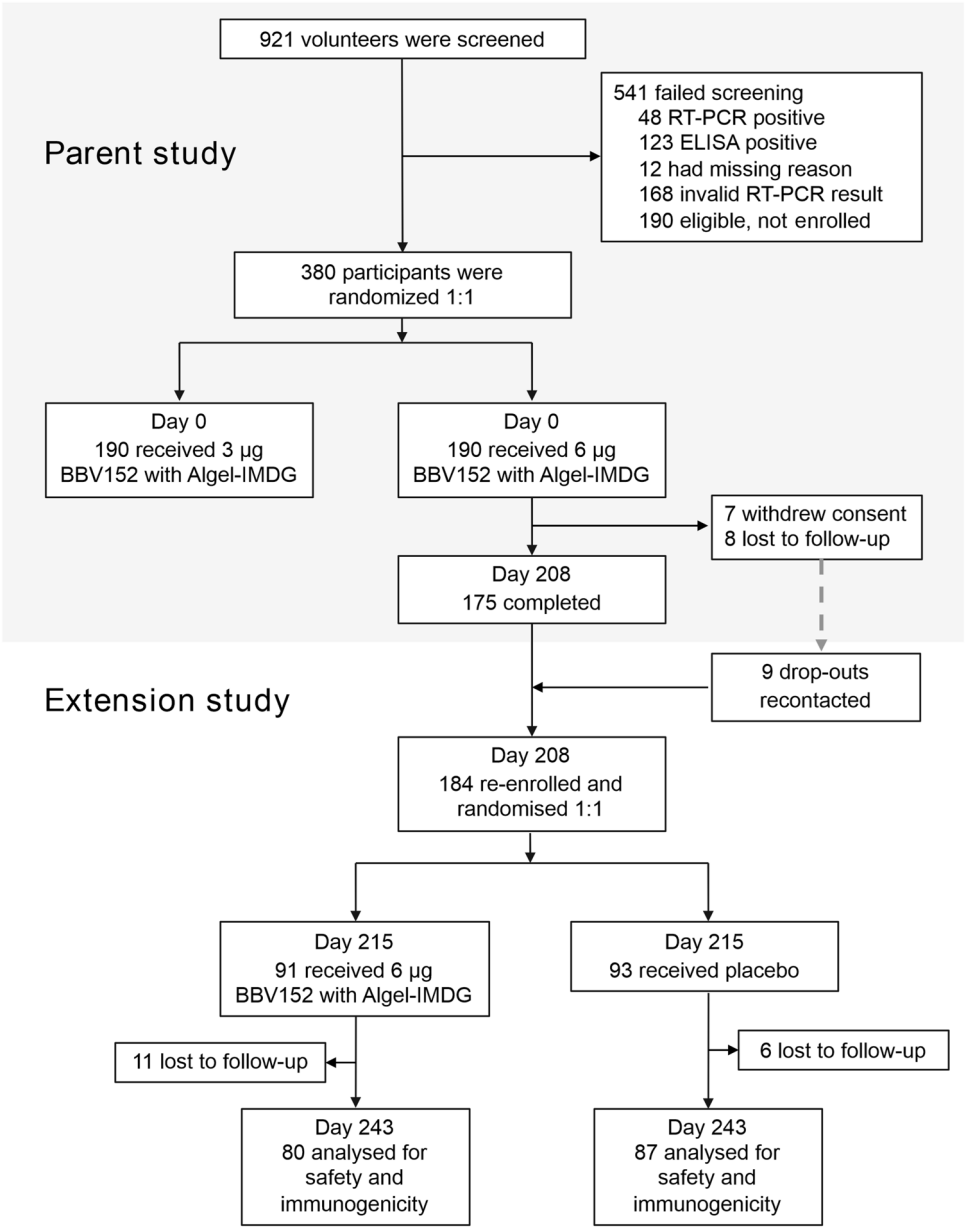
Study flow chart.

Of the 190 participants who originally vaccinated with the 6 µg BBV152 formulation with Algel-IMDG in the parent study between Sep 5 and Sep 12, 2020, 175 participants were still actively participating at Day 208, with 15 drop-outs (Fig. 1). On being recontacted, 9 of the 15 drop outs agreed to re-consent and participate in the extension study. Thus, a total of 184 participants were re-enrolled on Day 215 and randomized 1:1 to receive either a booster dose of BBV152 or placebo. Demographic characteristics of these participants are shown in Table 1.

**Table 1:** Demographics of participants enrolled in the booster dose study.

	Booster (N = 91)	Non-booster (N = 93)
**Age, years**		
Median	35.0 (25.0–44.0)	36.0 (26.0–44.0)
≥ 12 to < 18	0 (0%)	4 (4.3%)
≥ 18 to < 55	85 (93.4%)	82 (88.2%)
≥ 55 to ≤ 65	6 (6.6%)	7 (7.5%)
**Sex**		
Female	24 (26.4%)	20 (21.5%)
Male	67 (73.6%)	73 (78.5%)
Body Mass Index, kg/m^2^	25.3 (3.5)	24.7 (2.7)

Data represents median (IQR), n (%) or mean (SD).

As previously reported in the parent study two 6 µg doses of BBV152 administered 28 days apart induced high neutralizing antibody titers^11^. Thus, on Day 56, 4 weeks after the second dose, the PRNT_50_ GMT was 197 (95% CI 156–249) and the MNT_50_ GMT was 160 (136–189). Seroconversion was observed in 174 (98.3% [95% CI 95.1–99.7]) of 177 participants by PRNT, and in 171 (96.6% [92.8–98.8]) of 177 participants by MNT (Table 2).

**Table 2:** Neutralizing antibody titers against SARS-CoV-2 measured by PRNT and MNT.

		Geometric mean titer (95% CI)		Seroconversion rate^a^ (95% CI)	
		Booster	Non-booster	Booster	Non-booster
**Plaque reduction neutralization test (PRNT)**					
Parent study	Day 56	N = 177 197.0 (155.6–249.4)		174/177 98.3% (95.1–99.6)	
Booster study	Day 215	N = 91 23.6 (10.0–55.7)	N = 93 26.7 (11.0–64.6)	N = 91 67.4 (56.5–77.1)	N = 93 67.8 (57.1–77.3)
	Day 243	N = 80 746.2 (514.9–1081) p < 0.0001^b^	N = 87 100.7 (43.6–232.6) p < 0.0269^b^	N = 80 98.7 (92.8–99.9)	N = 87 79.8 (69.6–87.8)
**Microneutralization test (MNT)**					
Parent study	Day 56	N = 177 160.1 (135.8–188.8)		171/177 96.6% (92.8–98.8)	
Booster study	Day 215	N = 91 66.8 (49.7–89.7)	N = 93 85.0 (62.5–115.5)	N = 91 77.9 (67.7–86.1)	N = 93 80.0 (70.3–87.7)
	Day 243	N = 80 641.0 (536.8–765.3) p < 0.0001^b^	N = 87 359.3 (267.4–482.7) p < 0.0001^b^	N = 80 100 (95.3–100)	N = 87 92.9 (85.1–97.3)

PRNT GMTs against SARS-CoV-2 variants					
Variant	D614G	Alpha (B.1.1.7)	Beta (B.1.351)	Delta (B.1.617.2)	Delta plus (B.1.617.2 AY.1)
Day 208	N = 32 13.8 (4.5, 42.4)	N = 30 10.3 (2.8, 37.8)	N = 32 1.9 (0.5, 7.2)	N = 32 0.9 (0.2, 3.0)	N = 32 1.1 (0.3, 3.7)
Day 243	N = 37 669.4 (443.4, 1010)	N = 37 422.2 (278.4, 640.1)	N = 33 548.6 (373.6, 805.4)	N = 36 369.0 (221.7, 614.0)	N = 34 309.5 (198.4, 482.9)

^a^Defined as a post-vaccination titer that was at least fourfold higher than the baseline titer.

^b^P value between Day 215 and Day 243 values.

By the time of this follow up 6 months after the second dose, the levels of neutralizing antibodies had declined but persisted above baseline with GMTs on Day 208 of 23.9 (95% CI 14.0–40.6) by PRNT and 54.6 (41.7–71.5) by MNT; both values were significantly lower than the Day 56 values (both p < 0.0001). The majority of the 175 participants still had neutralizing antibody titers above baseline; 133 (76%) by PRNT and 155 (87%) by MNT. Following the decline in titers, we were able to estimate decay rate constants (k) of 0.006 and 0.003 based on PRNT_50_ and MNT_50_ titers, respectively (see Supplementary Fig. S1), while considering the peak titers on day 56. Based on the exponential decay model, the estimated half life of neutralizing antibody titers of all participants were predicted to be 50 days for PRNT_50_ and 98 days for MNT_50_.

Similarly, the GMT of specific binding IgG antibody titers against S-protein declined from 9542 (8246–11,041) on Day 56, to 2223.6 (1840.8, 2685.9) on day 208, but remained above the baseline with a fourfold seroconversion of 63.6% (55.5, 71.1). In similar pattern, anti-RBD and anti-nucleocapsid IgG antibodies gradually decline from Day 56 to Day 208, 2354.9 (1993.7, 2781.6 and 2334.2 (1965.9, 2771.5) with a fourfold seroconversion 69.5% (61.5, 76.5) and 66.9% (58.8, 74.1) respectively.

### Booster dose increased the magnitude of neutralization and binding antibody titers against D614G

Booster dose was administered on Day 215 and 4 weeks later (on Day 243), there were statistically significant increases in neutralizing GMTs, ~ 30-fold when measured by PRNT (746 [95% CI 515–1081], p < 0.0001) and ~ tenfold by MNT (641 [537–765], p < 0.0001). Seroconversion rates increased to 98.7% and 100%, respectively. The levels of neutralizing antibodies observed with both assays were higher after booster than than those observed after the two dose primary vaccination series (Table 2). GMTs and seroconversion rates also increased in the non-booster group, but to a lesser extent than the booster group. GMTs increased ~ fourfold, 101 (43.6–233) and 359 (267–483) for PRNT and MNT, respectively, with corresponding increases in seroconversion rates to 79.8% and 92.9% in non-booster group. The increase on Day 243 following booster vaccination was significantly higher than in the non-booster group when assessed by either PRNT or MNT assays (p < 0.005). Final MNT_50_ GMTs in both groups were statistically higher, p < 0.0001 for the booster arm and p < 0.005 for the non-booster arm, than the GMT of 128 (95% CI 65.0–250) observed for the 21 BEI reference sera. Gender wise comparision based on PRNT_50_ revealed that the neutralization antibody titers between males and females are comparable and there is no statistically significant difference (see Supplementary Fig. S2).

Similarly, the GMT of specific binding IgG antibody titers against S1-protein on Day 243 increased to 11,565 (9125–14,657) and 7109 (5316–9505), compared to GMTs on day 215, 4155 (2987–5781) and 5115 (3766–6948) in the booster and non-booster groupsrespectively (Table 3). Like-wise, the GMTs of anti-SARS-CoV-2 specific (RBD or N-protein) binding antibody titers were significantly higher in the booster group than the non-booster group (p value < 0.05) on day 243. The increase in anti-SARS-CoV-2 specific (S1-protein, RBD or N-protein) binding antibody titers in non-booster group are attributed to the natural exposure, as this study was conducted during the second wave of COVID -19 infections in India, when the delta variant was predominant^13, 14^.

**Table 3:** Binding IgG antibody titers against SARS-CoV-2 antigens measured by ELISA.

	Geometric mean titer (95% CI)		Seroconversion rate^a^ (95% CI)	
	Booster	Non-booster	Booster	Non-booster
**Spike (S1) protein**				
Day 56	n = 177 9542 (8246–11,041)		171/177 96.6% (38.9–60.0)	
Day 215	n = 91 4155 (2987–5781)	n = 93 5115 (3766–6948)	58/91 63.7% (52.9–73.4)	71/93 76.3% (66.2–84.3)
Day 243	n = 80 11,565 (9125–14,657)	n = 87 7109 (5316–9505)	74/80 93.8% (86.0–97.9)	72/87 81.6% (71.9–89.1)
**Receptor binding domain (RBD)**				
Day 56	n = 177 5558 (48,606–63,571)		167/177 94.4% (89.9–97.3)	
Day 215	n = 91 3135 (2299–4274)	n = 93 3825 (2826–5177)	58/91 63.7% (52.9–73.4)	67/93 72.0% (61.6–80.6)
Day 243	n = 80 7013 (5483–8971)	n = 87 4294 (3223–5721)	71/80 89.8% (79.7–94.7)	65/87 74.7% (64.3–83.4)
**Nucleocapsid (N) protein**				
Day 56	n = 177 8754 (7589–10,097)		171/177 96.6% (92.8–98.8)	
Day 215	n = 91 3281 (2422–4446)	n = 93 3970 (2921–5396)	60/91 65.9% (55.2–75.3)	63/93 67.7% (57.1–76.9)
Day 243	n = 80 8359 (6461–10,815)	n = 87 5147 (3768–7030)	74/80 92.5% (84.4–97.2)	65/87 74.7% (64.3–83.4)

^a^Defined as a post-vaccination IgG titer that was at least fourfold higher than the baseline titer.

### Booster dose increased the neutralization efficiency against both homologous and heterologous variants (VOCs)

Neutralizing antibody GMTs against the SARS-CoV-2 variants (Alpha, Beta, Delta and Delta plus) were assessed by PRNT on Days 208 and 243 in the booster group; no samples were analysed in non-booster recipients. At Day 243 the GMT against the BBV152 strain (D614G) in this group was 669 PRNT_50_ (95% CI 380–1052). As shown in Table 2 GMTs against Alpha, Beta, Delta and Delta plus variants were 422 (278–640), 549 (374–805), 369 (222–614) and 310 (198–483) following 49-, 41,- 289-, 410- and 281-fold rises from Day 208, respectively (See Supplementary Fig. S3). The GMT ratio between the BBV152 strain-D614G *vs*. the Delta variant was 2.0 (95% CI 1.4–2.8) on day 243. Further, we also showed 18.5-fold increase in neutralization antibody titers against Omicron variant after booster dose of BBV152^15^, compared with non-booster recipients. Moreover, 90% of subjects, who received third dose of COVAXIN^®^ showed neutralizing activity against the Omicron variant, when tested with 28 days, post third dose sera^16^.

In addition, sera collected after 6 months post thrid dose were also evaluated for neutralization efficiency against D614G, Delta and Omicron by PRNT_50_ assay. Geometric mean titers (GMTs) were found to be above the baseline on Day 395 in both the arms, though the decline of antibodies noticed from Day 243. However, 93.5–96.8% boosted subjects showed neutralizing activity with fourfold seroconversion against the D614G, Delta and Omicron variant in booster arm, while the non-booster arm showed 56.8–59.5% seroconversion (Table 4). These findings showed that booster dose of COVAXIN^®^ generated higher neutralization efficiency against D614G, Delta and Omicron Variant and the antibody titers are persistent even after 12 months of primary vaccination series.

**Table 4:** PRNT_50_ GMTs against SARS-CoV-2 variants (12 months post 2nd dose or 6 months post third dose, day 395).

	Geometric mean titer (95% CI)		Seroconversion rate^a^ (95% CI)	
	Booster	Non-booster	Booster	Non-booster
D614G (NIV-770-2020)	N = 31 178.9 (82.6–387.5)	N = 37 10.7 (2.6–44.5)	30/31 96.8% (81.5–99.8)	22/37 59.5% (42.2–74.8)
Delta (B.1.617.2)	N = 31 115.9 (55.8–240.8)	N = 37 7.3 (2.0–27.0)	30/31 96.8% (81.5–99.8)	22/37 59.5% (42.2–74.8)
Omicron (B.1.1.529)	N = 31 25.7 (13.0–50.6)	N = 37 2.9 (0.99–8.3)	29/31 93.5% (77.2–98.9)	21/37 56.8% (39.6–72.5)

^a^Defined as a post-vaccination IgG titer that was at least fourfold higher than the baseline titer.

### Th1 biased immune response is sustained after the booster dose

Th1 and Th2 dependent immunoglobulin subclasses (IgG1 and IgG4, respectively) measured by ELISA on Days 215 and 243 showed Th1-biased response in both booster and non-booster groups (see Supplementary Fig. S4), as reported earlier^11, 12, 17^. On Day 215, the median Th1:Th2 index in booster group was 10.0 (IQR, 1.0–32.0) which increased to 16.0 (IQR, 4.0–32.0) on Day 243, with higher titers of IgG1 than IgG4.

### Vaccine induced IFNγ T cell responses persisted, at 12 months, post second dose

SARS-CoV-2-specific IFNγ release was evaluated using a whole blood T-cell immunity assay from a subset of vaccinated participants on Day 208, (6 months after the second dose). The median IFN-γ release obtained from SARS-CoV-2 stimulated T cells of vaccinated subjects was 148.8 ng/ml, which is statistically significantly higher (p < 0.0001) than the corresponding unstimulated cells, 26.6 ng/ml (see Supplementary Fig. S5). This indicates the persistence of T cell responses up to 6 months after the second dose.

On Day 395 (6 months post 3rd dose or 12 months post 2nd dose), SARS-CoV-2 specific IFNγ secreting cells were analyzed by ELISpot assay between the booster and non-booster group. The SARS-CoV-2T cell recall responses were found similar in both the arms with a median 48 (15.0–85.0) and 48 (29.0–95.0) in booster and non-booster group respectively (Fig. 4).

### Presence of central and effector memory T cells phenotype demonstrated the significant antigen recall responses against antigen re-exposure

**Figure 2:**
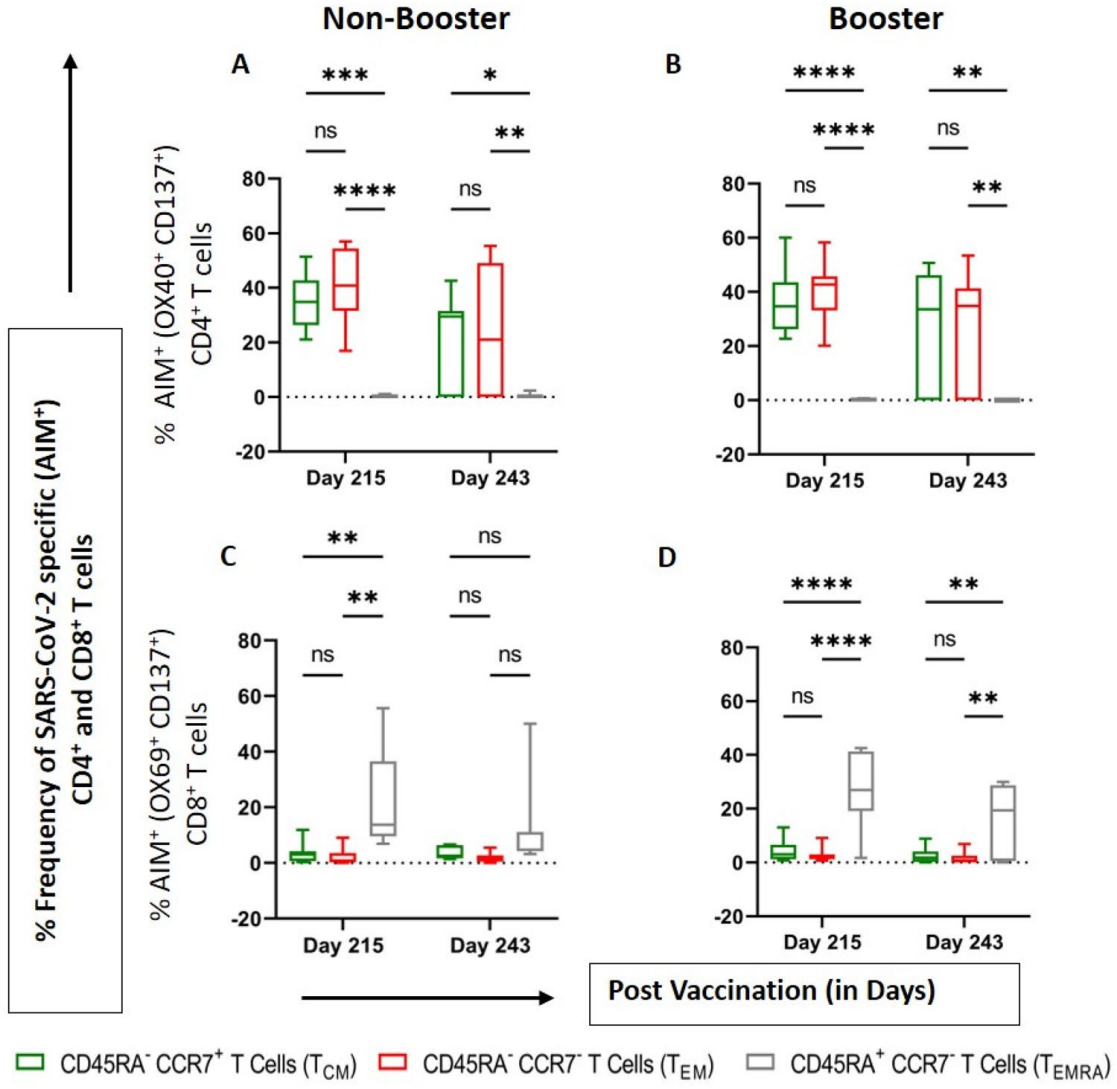
Persistence of T and B cell memory responses against SARS-CoV-2 (D614G). Boxes display the SARS-CoV-2-specific T and B cell memory responsesafter two doses of BBV152 on Days 0 and 28 measured on Day 215 (before booster dose) and Day 243 (28 days after the booster dose of BBV152). PBMC samples collected from 15 participants (n = 8 non-booster and n = 7 BBV152 recipients). Boxes indicate upper and lower quartiles,lines within the box indicate median and whiskers extending from the boxes indicate the upper and lower quartiles.*p < 0.05, **p < 0.01, ***p < 0.005 and ****p < 0.0001.

T cell responses were assessed by activation-induced marker (AIM) assay in PBMCs stimulated with overlapping peptide pools of SARS-CoV-2 S, M, and N proteins. CD137^+^OX40^+^ and CD137^+^CD69^+^ cells were considered SARS-CoV-2-specific cells among CD4^+^ and CD8^+^ T cells respectively. Vaccine-induced SARS-CoV-2 recall responses were demonstrated by the presence of SARS-CoV-2-specific CD4^+^ or CD8^+^ central (CCR7^+^CD45RA^−^, T_CM_), effector (CCR7^−^CD45RA^−^, T_EM_) and Terminally differentiated effector memory cells that re-express the CD45RA(CCR7^−^CD45RA^+^, T_EMRA_) T cell population. On day 215, among the SARS-CoV-2-specific CD4^+^ T cell population,the proportions of T_CM_ were 34.6% and 34.8% in booster and non-booster groups, respectively, and the proportions of T_EM_ cells were 42.7% and 40.8%, whereas proportions of CD4^+^ CCR7^−^CD45RA^+^ (T_EMRA_) cells were minimal, 0.2% and 0.0% (Fig. 2) in both groups. In contrast, among the SARS-CoV-2-specific CD8^+^ T cell populationat Day 215 the proportions of T_EMRA_ cells were 26.9% and 13.7% in booster and non-booster groups, while the proportions of T_CM_ and T_EM_ cells were minimal (Fig. 2). On Day 243, vaccine-induced SARS-CoV-2-specific CD4^+^ or CD8^+^ recall memory responses were similar to Day 215 in all the memory phenotypes tested. Collectively, these results demonstrate a phenotypic profile of antigen specific CD4^+^and CD8^+^T cells associated with protective immunity to SARS-CoV-2 infection with a good antigen recall response.

### Booster dose increases memory B cell response with an increase of IgG secreting B cells

To evaluate antigen-specific memory B-cells (MBC), PBMCs were stimulated for polyclonal stimulation followed by stimulation with inactivated SARS-CoV-2 antigen. The ability of MBCs to differentiate into antibody-secreting cells (ASC) upon polyclonal stimulation, was measured as a readout of humoral immune memory in the ELISpot assay^18^.

**Figure 3:**
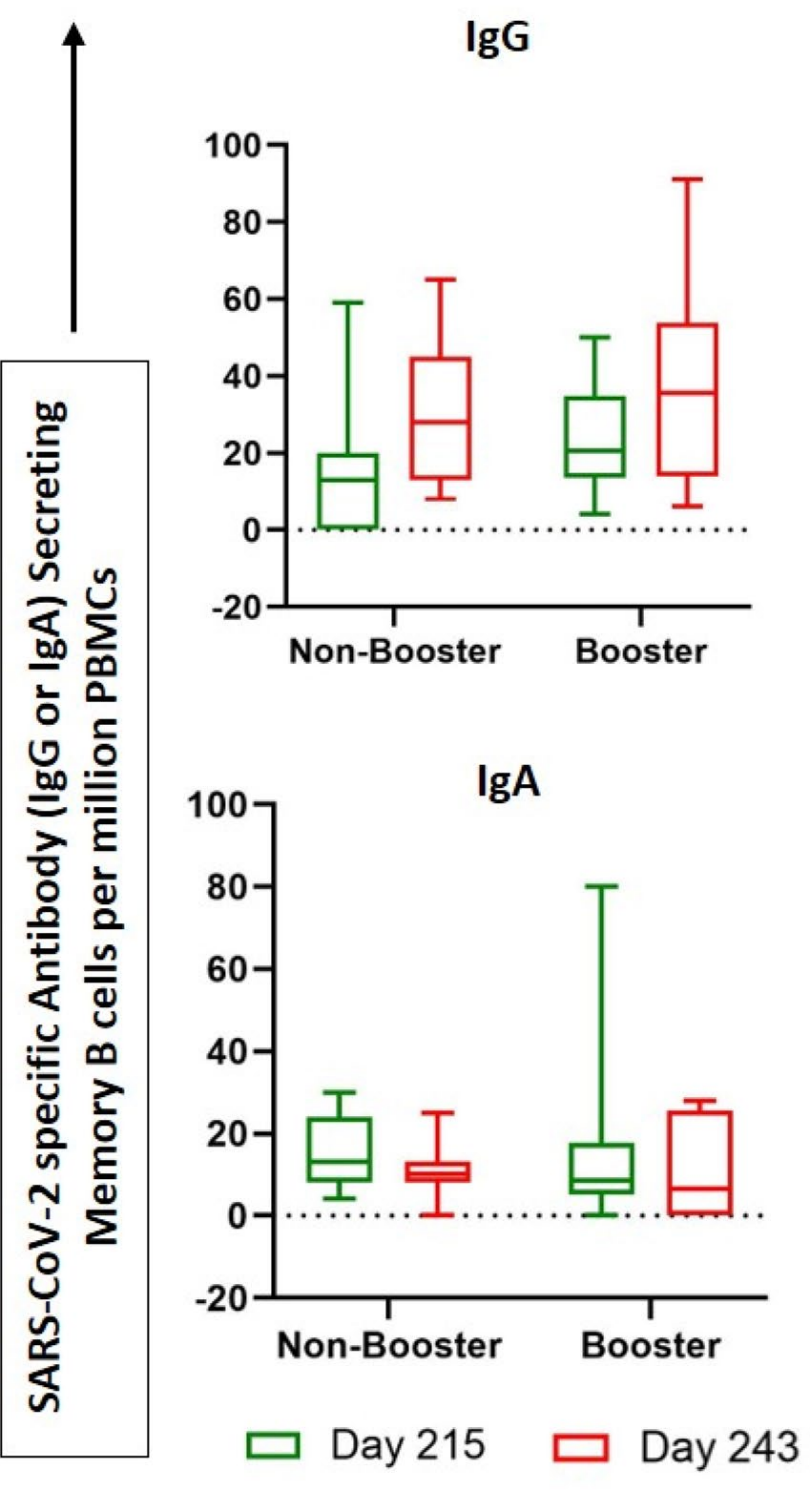
Vaccine-induced antigen-specific antibody (IgG and IgA) secreting memory B-cell responses performed by ELISpot assay. PBMCs collected from vaccinated subjects on Day 215 were pre-activated or pre-stimulated with polyclonal expansion using Poly B stimulant for 4 days, with unstimulated cells or cells without pre-activation as negative controls. Antibody-secreting cells (ASC) were detected with anti-human IgG (biotin) and anti-human IgA (FITC) antibody followed by streptavidin–ALP and FITC-HRP respectively. Boxes indicate upper and lower quartiles, lines within the box indicate median and whiskers extending from the boxes indicate the upper and lower quartiles.

**Figure 4:**
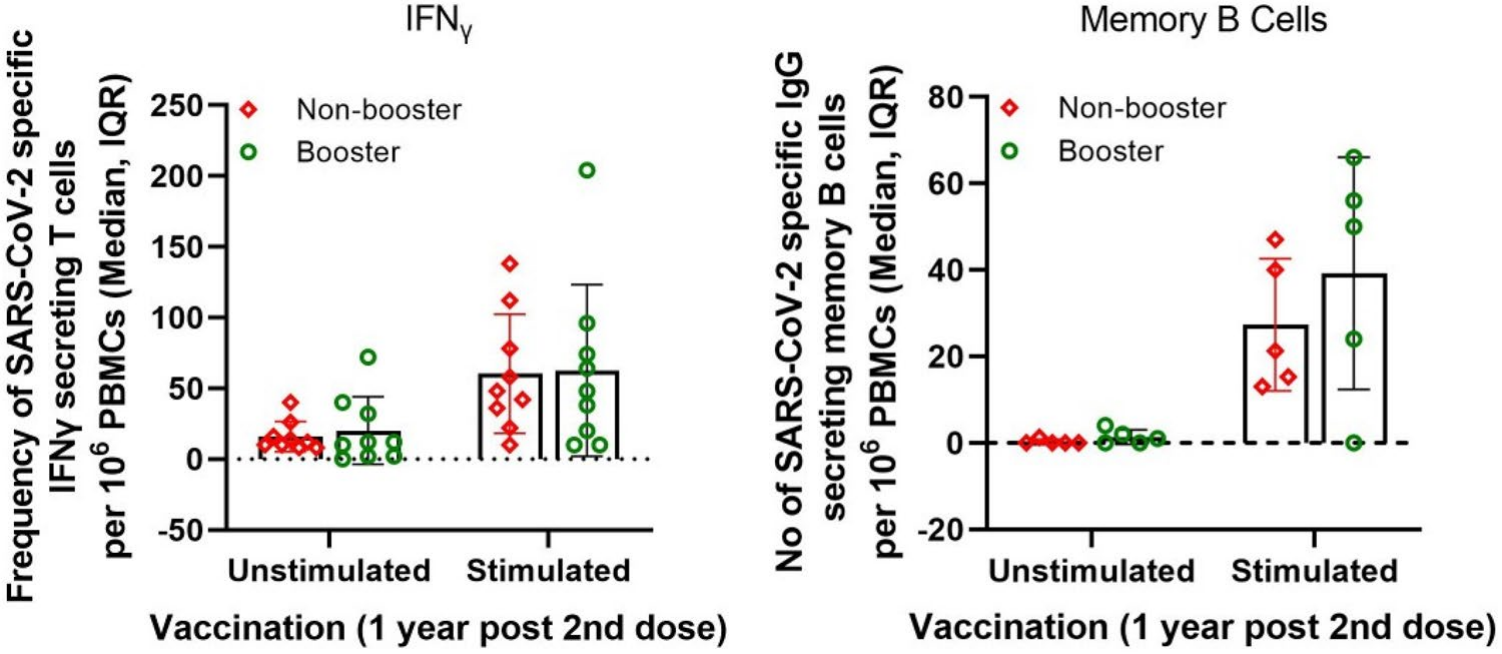
Persistence of SARS-CoV-2 antigen recall T cell responses until 12 months post 2nd dose and increased memory B cell response with the booster dose. PBMCs collected on Day 395 were used for both the assays.

There was persistence of long-lived memory B cells, demonstrated by the detectable levels of SARS-CoV-2 specific IgG and IgA secreting B cells from both booster and non-booster groups (Fig. 3), on Days 215 and 243. On Day 215, the median numbers of SARS-CoV-2 specific antibody (IgG) secreting memory B cells (MBC) per 10^6^ PBMCs were 20.5 (IQR 13.5–34.8) and 13.0 (0.0–20.0) in booster and non-booster groups, respectively. These numbers increased on Day 243 to 35.5 (14.0–53.8) and 28.0 (13.0–45.0) in booster and non-booster groups. On Day 215, the median numbers of SARS-CoV-2 specific antibody (IgA) secreting memory B cells (MBC) per 10^6^ PBMCs were 8.5 (IQR: 5.0–17.8) and 13.0 (8.0–24.0) in booster and non-booster groups, respectively, with small reductions on Day 243 to 6.5 (0.0–25.5) and 10.0 (8.0–13.0) in booster and non-booster groups, respectively. Similarly, 6 months post third dose, MBC response, IgG secreting memory B cells (MBC) per 10^6^ PBMCs was median 50 (12.0–60.0, IQR) remains elevated in booster arm over the non-booster arm with a median 21.3 (14.2–43.5, IQR) (Fig. 4).

### Booster dose of BBV152 is safe without serious adverse events

After the third dose of BBV152, there were 8 solicited adverse events in the booster groups and 5 in the non-booster group. All 8 in the booster group were local reactions at the injection site, 5 (5.4%) cases of pain, 2 (2.1%) of itching and one case of redness (1.0%). In the non-booster groups the 5 reports consisted of 2 (2.1%) cases of injection site pain, 2 (2.1%) of fever and 1 (1.0%) instance of headache (see Supplementary Table S2). Most of these adverse events were described as mild and resolved within 24 h of onset. No unsolicited adverse events or symptomatic SARS-CoV-2 infections were reported to investigators through telephone follow-up or site study visits between Day 0 and the scheduled visit on Day 243. Additional telephone calls were conducted to ensure complete documentation of any breakthrough infection, but routine RT-PCR for SARS-CoV-2 was not done. No serious adverse events, including hospitalisation and death,were reported through Day 243.

## Discussion

We show that two doses of BBV152 (6 µg with Algel-IMDG) elicited durable neutralizing antibody responses until 6 months after the second dose together with persistent T cell and B cell responses. There was a decline of antibody levels after the second dose of vaccine in concordance with earlier reported literature^19, 20^. However, more than 75% of all participants followed up 6 months post second dose still had detectable neutralizing antibody responses to the homologous vaccine SARS-CoV-2 strain (D614G). Another study, comparing COVAXIN^®^ with Covishield also showed that there was significant decline in antibody titers in Covishield recipients compared to COVAXIN^®^ recipients at 6-months^21^. Further, 28 days after a booster dose of BBV152 there was a marked increase in titers to higher levels than those achieved after the two-dose primary series. When assessed against heterologous strains representing the predominant Variants of Concern, humoral immunogenicity increased (40- to 410-fold). Neutralizing antibody titers were comparable to an internationally accepted reference panel of convalescent sera. We also demonstrated significantly elevated neutralizing antibody responses, against Omicron the currently predominant circulating variant, after the third dose of BBV152. These results are quite encouraging and provides assurance of a protective immune response against Omicron^15, 22^.

Booster vaccination was well tolerated with few adverse events, the most frequent being mild and transient pain and itching at the injection site. No severe or life-threatening solicited adverse events were reported, and no significant safety differences were observed between BBV152 and control groups; nor were there any safety concerns raised when reactogencity of the booster was compared with the primary vaccination series. Although the study was not powered to compare such differences, the combined incidence rate of local and systemic adverse events after any dose of BBV152 is noticeably better than the rates for other SARS-CoV-2 vaccine platform candidates^23–26^ and comparable to rates observed for other inactivated SARS-CoV-2 vaccine candidates^27, 28^. However, other vaccine studies enrolled different populations and employed varying approaches to measure adverse events. Additional safety data has also been obtained for COVAXIN^®^, administered as Booster/Precautionary dose to around 2 million individuals of > 60 years of age, from the post-marketing surveillance through CoWIN app, Government of India. Only one AEFI recorded along with other AEs which were mild in nature and got resolved. This establishes COVAXIN^®^ is tolerable as a booster dose.

As previously reported BBV152-induced antibodies from sero-negative individuals, showed no significant decrease in neutralization activity against the Alpha (B.1.1.7) variant, but demonstrate marginal reductions in neutralization activity, by 2-, 2-, 3-, and 2.7-fold, respectively, against the B.1.1.28, B.1.617.1, B.1.351 (Gamma), and B.1.617.2 (Delta) variants^29–32^. Here, we report lower neutralization activity against wild type SARS-CoV-2 and VOCs on Day 208, 6 months after the second vaccination, but following a third dose of BBV152, serum neutralizing antibody titers demonstrated 48.5-fold and 410-fold increases against D614G (the BBV152 vaccine strain) and the Delta variant, respectively. The PRNT GMT ratio for D614G to Delta was 2.0 (95% CI 1.4–2.8) indicating a comparable neutralization profile after three doses of BBV152.

Durable and persisting immune memory against SARS-CoV-2 has been noted after natural infection^33–35^. Similarly, we found a pronounced SARS-CoV-2-specific T cell response to BBV152, with a majority of CD4^+^ T central (CD4^+^ T_CM_) and effector phenotype (CD4^+^ T_EM_), including distinct CD8^+^ T_EMRA_ phenotype, before and after the booster dose with a good antigen memory responses. This may allow BBV152 to confer long term protective efficacy against SARS-CoV-2 variants. Vikkurthi et al.^36^ have shown the potential of BBV152 to induce spike and nucleospecific circulating Tfh cells—Tfh1 (CXCR3^+^CCR6^−^), Tfh2 (CXCR3^−^CCR6^−^) or Tfh17 (CXCR3^−^CCR6^+^)—that help in B cell production. Distinct CD8^+^ T_EMRA_ phenotype induced by BBV152 could be attributed to the memory response against conserved nucleoprotein, indicating the nucleoprotein as a potential target for SARS-CoV-2 vaccines. As the current VOCs show major mutations in the viral spike protein, if BBV152 vaccine induces immune memory against conserved nucleoprotein it may provide an additional advantage to protect against immune escape variants. In addition, a small increase in the levels of memory B cells after the booster was correlated with an increase in the neutralization potency against both homologous and heterologous strains which supports vaccine-induced memory B cells playing a role in protection against circulating SARS-CoV-2 variants.

An unexpected observation was the small increase in neutralizing GMTs between Days 215 and 243 in those who received placebo (non-booster) rather than a booster dose. This is most likely to be due to natural infection as this booster study was conducted during the second wave of COVID-19 progression in India which was dominated by the Delta (B.1.617.2) SARS-CoV-2 variant and peaked between March 29, 2021 and July 6, 2021 (see Supplementary Fig. S6). There were also increases in SARS-CoV-2-specific IgG binding antibodies in some individuals further supporting the suggestion of natural infection of these individuals. However, none of the 380 participants who enrolled in the parent Phase 2 study reported any of the adverse events expected to be associated with COVID-19, nor were there any deaths or hospitalisations through to the end of this booster study.

It is interesting that despite the dominance of Delta variant during this period there were no cases of COVID-19 detected in the study population. In an efficacy trial BBV152 displayed 65.2% (95% CI 33.1–83.0) protection against the SARS-CoV-2 Delta VOC^12^. Real world effectiveness of 50% (95% CI 33–62) and 57% (21–76) after two doses administered at least 14 and 42 days apart was demonstrated against Delta in a cohort of healthcare workers known to be exposed to higher infectious pressure^37^. BBV152 has been shown to produce broad specific cell mediated responses against several VOCs^31^. Collectively, these results suggest BBV152 induced T cell and B cell memory responses to protect against infection although the SARS-CoV-2-specific antibody responses did decline over the 6 months post dose 2. SARS-CoV-2 specific memory T cell responses have been reported to remain detectable in convalescent individuals up to 10 months after infection^34^, and one study found that administration of a third dose of CoronaVac 6 months after a second dose was effective in recalling SARS-CoV-2-specific immune responses, with a marked increase in antibody levels^38^. However, unlike ours, that study did not report on persistence of cell mediated responses at 6 months after the second dose.

Our results do not permit efficacy assessments, although we have demonstratetd efficacy of BBV152 in a larger study^12^. The present study enrolled a limited number of participants aged from ≥ 12 to ≤ 65 years, and further studies will be required to establish the effect of a booster dose in the elderly or immunocompromised. An ongoing longitudinal follow-up of additional post-vaccination visits (months 9 and 12) will be important to understand the durability of immune responses. However, this study is the first double-blind, randomized, controlled trial to evaluate both humoral and cell-mediated responses to a SARS-CoV-2 booster dose vaccine. In the parent study, 6 months after the second dose of BBV152, we observed durable neutralizing antibody responses that were significantly higher than an international reference serum panel.

We have previously reported an interim efficacy of 93% against severe COVID-19 and 65% against any severity of disease due to the Delta variant (median follow-up of 4.7 months after dose 1). This report contains data on humoral and cell-mediated response from a booster dose of BBV152. No clinical endpoints were evaluated and the level of neutralizing antibodies after the third dose cannot be used to infer any level of protection, although there is data to suggest a correlation between neutralizing antibody titers to protection^39^. The Delta variant has been reported to have a shorter incubation period (4 days) compared with the ancestral Wuhan (6 days)^40^ so faced with a decline in neutralizing antibodies, persistent cell mediated memory responses maybe important to provide durable vaccine efficacy against severe COVID-19^35^. However, a marked reduction in efficacy against breakthrough infections leading to mild to moderate disease may be expected, and the long term efficacy of BBV152 against severe COVID-19 is currently being evaluated in a phase 3 study. With the earlier demonstration of 65% efficacy against Delta at 4.7 months median follow-up, and a favourable reactogenicity profile of an inactivated vaccine, a broad antibody response to a third booster dose may be advised to ensure robust neutralizing antibody titers that prevent breakthrough variant-related mild to moderate disease and infection^41^.

In conclusion, the presence of recalled T and B cell responses to SARS-CoV-2 specific antigen stimulation, 6 months after a 2dose vaccination schedule suggests good immune memory and effectiveness of vaccine against homologous SARS-CoV-2 strain. However, the marked increase in neutralizing titers against both homologous and heterologous strains (Alpha, Beta, Delta, Delta plus and Omicron) with a three dose regimen may necessitate roll out of booster vaccination to provide immune protection against new emerging SARS-CoV-2 variants.

## Methods

### Clinical trial design and participants

The parent study was a randomized, double-blind, multicentre phase 2 trial to evaluate the immunogenicity and safety of a whole-virion inactivated SARS-CoV-2 vaccine (BBV152) in healthy male and female volunteers in nine Indian hospitals^11^. The trial protocol was approved by the National Regulatory Authority (India) and by the respective hospital Ethics Committees (PGIMS, Haryana; AIIMS, New Delhi; Jeevan Rekha, Belgaum; Gillukar Multispeciality Hosptital, Nagpur; AIIMS, Patna; SRM Hospital & Research Center, Kattankulathur; NIMS, Hyderabad; Prakhar Hospital, Kanpur; Redkar Hosptial, Goa—see Supplementary Table S1) and the trial conducted in compliance with all International Council for Harmonization (ICH) Good Clinical Practice guidelines. The protocol is registered with the Clinical Trials Registry (India) No. CTRI/2021/04/032942, dated 19/04/2021 and on Clinicaltrials.gov: NCT04471519.

Participants were ≥ 12 to 64 years of age at the time of enrolment, and were negative for both SARS-CoV-2 nucleic acid and serology tests at baseline (before receiving the primary vaccination series). A total of 380 participants had been enrolled and randomized 1:1 to receive two doses of BBV152 formulations containing either 3 µg or 6 µg doses of antigen with Algel-IMDG administered on Days 0 and 28. They were followed up for 6 months after dose 2 (Day 208) to evaluate the persistence of safety and immunogenicity^11^.

Following an amendment to the protocol, 6 months after dose 2, a new informed consent was obtained from participants who originally received the 6 µg dose of BBV152, including some who had “dropped out” of the parent study. These participants were then randomized 1:1 to receive either a third (booster) dose of vaccine or placebo (on Day 215). Randomization, using a block size of four, was done by Interactive Web Response System at contract research organisation (Sclin Soft Technologies). Participants, investigators, study coordinators, study-related personnel, and the sponsor were masked to the treatment group allocation.

### Clinical trial vaccine

BBV152 is a whole-virion ß-propiolactone-inactivated SARS-CoV-2 vaccine adjuvanted with Algel-IMDG. The virus strain NIV-2020-770 was isolated from a COVID-19 patient, sequenced at the Indian Council of Medical Research-National Institute of Virology (NIV), and provided to Bharat Biotech International limited (BBIL)^29, 42–45^. The vaccine virus strain NIV-2020-770 contains the D614G mutation, which is characterised by an aspartic acid to glycine shift at amino acid position 614 of the spike protein^42^. The control arm received non-booster containing sterile phosphate-buffered saline and Algel-IMDG. Vaccine and control formulations were supplied as 0.5 mL doses in single-use glass vials which were stored between 2 and 8 °C. The appearances of vaccine and non-booster were identical. Vaccine and non-booster were administerd by intramuscular injection in the deltoid muscle.

### Outcomes

The primary endpoints were neutralizing antibody titers against wild-type virus evaluated by two neutralization assays; a plaque-reduction neutralization test (PRNT) and a microneutralization assay (MNT) done at BBIL as previously described^11, 12, 44^. Secondary endpoints were the percentages of participants with solicited local reactions and systemic adverse events occurring within seven days after vaccination. Neutralizing responses against SARS-CoV-2 variants and cell-mediated immune responses were evaluated as exploratory endpoints.

### Clinical trial monitoring and sample collection

In the extension study, blood sample was collected on Day 215 (before administration of vaccine or non-booster) and on Day 243 (28 day, post 3rd dose) to determine antibody titers. Additional blood volume (10 mL) was collected with informed consent from a subset of participants from each group, on Days 215 and 243, to isolate peripheral blood mononuclear cells (PBMCs).

Participants were observed for 2 h post-vaccination to assess immediate reactogenicity. They were instructed to record solicited, unsolicited, local reactions and systemic adverse events within seven days of the booster dose using a paper-based memory aid and it was collected during the next visit to the site. Routine telephone calls were scheduled during the first 7 days after vaccination to ensure compliance.

Solicited local adverse events included pain and swelling at the injection site, and systemic adverse events included fever, fatigue/malaise, myalgia, body aches, headache, nausea/vomiting, anorexia, chills, generalised rash, and diarrhoea. Any unsolicited adverse events were reported by participants throughout the study. The investigator graded adverse events according to the severity score (mild, moderate, or severe) and whether they were related or unrelated to the investigational vaccine, as detailed in the protocol. At a follow-up visit on Day 243 safety data were collected and a second blood sample was drawn for immunogenicity assessments. All methods were performed in accordance with the relevant guidelines and regulations.

### Live virus neutralization assays

Neutralizing antibody titers against wild type virus were measured by live virus neutralization assays by both plaque-reduction neutralization test (PRNT) and microneutralization Test (MNT), at BBIL as described earlier^11, 12, 17, 44^ and a subgroup of serum samples were assessed for neutralizing antibody titers against both homologous (NIV-770-2020, D614G) heterologous strains (Alpha, Beta, Delta and Delta plus) by PRNT at NIV^15^. As there is no established SARS-CoV-2 correlate of protection, vaccine-induced responses were compared with an internationally recognised reference serum panel (BEI, Biodefense and Emerging Infections Research Resources Repository, NIAID, NIH, USA).

#### Enzyme linked immunosorbent assay (ELISA)

IgG-binding antibody binding titers were determined by ELISA, as previously described^11, 12, 44^ against the SARS-CoV-2 specific proteins such as S1, RBD and N protein.

#### Activation-induced marker (AIM) assay

Activation Induced marker assay was performed as reported earlier^30^. Briefly, PBMCs (0.7 million cells/100 µl) were plated onto separate 96 well U bottom plates and stimulated with the cocktail of SARS-CoV-2 peptide pool (1 µg/mL). After overnight stimulation, cells were washed and stained 40 min with antibody cocktail containing the following fluorescently conjugated antibodies obtained from Biolegend, USA: CD3-APC-A750 (300470), CD4-PB450 (300521), CD8a-APC-A700 (301028), OX40-APC (350008), CD137-ECD (309826), CD69-PC7 (310912), CCR7-PE (353204), CD45RA-FITC (304148). The plates were centrifuged and the resuspended cells were labelled again with 7AAD solution (6604104, Beckman) and analysed in a flow cytometer (CytoFlex S, Beckman Coulter).

#### Human IgG/IgA double colour Enzymatic ELISpot Assay

Human IgG/IgA double color Enzymatic ELISpot Assay was performed as per instruction manual (*IgAIgG-DCE-2M/10,* CTL). Briefly, human PBMCs were revived and resuspended in RPMI complete media and stimulated with 1:1000 of polyclonal B (Poly-B) cell activator solution (CTL-hBPOLYS-200, CTL) for 4 days at 37 °C. On Day 3, activated ELISPOT plate was coated with SARS-CoV-2 antigen for overnight at 4 °C. On Day 4, the plate was blocked and added with PBMC’s (0.3 × 10^6^ cells/well), kept for incubation at 37 °C, with 5% CO_2_ for 16–18 h. On Day 5, the plate was washed and added diluted detection solution of Anti-human IgA (FITC) and Anti-human IgG (Biotin) and incubated at RT for 2 h. After suffiecient washes, tertiary solution containing FITC-HRP and SA-AP added and incubated at RT (in dark condition) for 1 h. Spots were developed by the addition of TrueBlue or TrueRed for the visuallization of IgG and IgA secreting cells respectively. Assay controls, unstimulated cells and cells stimulated with Influenza antigen were maintained. Plates dried and read with help of ELISPOT reader.

#### Whole blood T-cell immunity assay kit for COVID-19

Whole blood T-cell immunity assay is performed as per manufacturer instructions (Immunitas BioSciences, Bangalore). Briefly, whole blood is centrifuged and the cell pellet was resuspended in T cell assay nutrient media. Cells (500 µl) transferred into three vials (test, positive and negative control) provided along with the kit. After gentle mixing, vials incubated for 18–20 h with 5% CO_2_ at 37 °C. Post incubation, vials spun and supernatant is used to perform IFNγ ELISA using the kit provided by the manufacturer.

#### ELISpot assay

ELISPOT assay was performed using the IFN-γ ELISPOT kit (MABTECH), as per the manufacturer’s instructions. The PBMCs collected on Day 395 from both booster and non-booster arm were used and stimulated with SARS-CoV-2 peptide matrix (SARS-CoV-2 S, M & N).

#### SARS-CoV2 spike (S1) antibody (IgG1/IgG4) isotyping

Th1-dependent IgG1 *vs*. Th2-dependent IgG4 antibody subclasses were determined by ELISA as described previously^9, 40^. Briefly, 96 well microtiter plates were coated with spike (S1) protein (SYNG-PRB026913, Syngene), at a concentration of 1 µg/ml, in PBS pH 7.4) and blocked with 1% BSA in PBS, pH 7.4. Twofold serially dilutions of individual sera (1:50 to 1:6400) were added and incubated for 2 h at 37 °C followed by the addition of mouse anti Human IgG1 HRP (A-10648, Invitrogen) or IgG4 (A-10654, Invitrogen) antibodies at a dilution, 1:1000. After incubation, TMB solution (AR1002, deNovo Biolabs) used as a substrate followed by the stop solution. Absorbance read at 450 nm. End point antibody titers determined and Th1:Th2 index was calculated as described previously^9, 40^.

### Statistical analysis

No formal sample size estimation was made to compare differences in neutralizing antibody responses between the booster and control arm. Safety endpoints are presented descriptively as frequencies (%) per group. Immunological endpoints and group seroconversion rates are presented as group GMTs with 95% confidence intervals (CIs); seroconversion was defined as post vaccination titer ≥ fourfold above the pre-vaccination titer in each participant. For continuous variables (below 20 observations), medians and IQRs are reported. The exact binomial calculation was used for the CI estimation of proportions. Wilson’s test was used to test differences in proportions. The GMT confidence intervals were estimated based on the log_10_ (titer) and the assumption that the log_10_ (titer) was normally distributed. A comparison of GMTs was performed with t-tests on the means of the log_10_ (titer). Significance was set at p < 0.05 (2-sided). Descriptive and inferential statistics were performed using SAS 9.2. For the other T and B cell immune responses, statistics were performed using Graph pad prism, version 8.0 and R version 4.1.2.

### Reporting summary

Further information on research design is available in the Nature Research Reporting Summary linked to this article.

## Supplementary Information

Supplementary Information 1.

Supplementary Information 2.

## Data Availability

SARS-CoV-2 strain (NIV-2020-770) sequence was deposited in the GISAID (GenBank: EPI_ISL_420545). The authors also declare that the data supporting the findings of this study are available within the main and supplemental figures. Individual participant (de-identified) data will be made available when the trial is complete upon a direct request to the corresponding author with an appropriate research proposal. After consideration and the approval of such a proposal data will be shared through a secure online platform.

## Code availability

No new code was generated in this study.
