# Supplementary material for: Persistence of immunity and impact of third dose of inactivated COVID-19 vaccine against emerging variants
Source: Sci Rep. 2022 Jul 14;12:12038. doi: 10.1038/s41598-022-16097-3 (PMC9281359; doi:10.1038/s41598-022-16097-3)
Supplement: Supplementary file 1 — Supplementary Information 1. [file 41598_2022_16097_MOESM1_ESM.pdf]

Supplementary Information for

Persistence of immunity and impact of third dose of inactivated COVID-19 vaccine against emerging variants

Krishna **Mohan**Vadrevu, PhD [1*], Brunda **Ganneru**, PhD [1],Siddharth **Reddy**, MSc [1], Harsh **Jogdand**, DVM[1], Dugyala **Raju**, PhD [1], Gajanan **Sapkal**, PhD [2], Pragya **Yadav**, PhD [2], Prabhakar **Reddy**, MD [3], Savita **Verma**, MD [4], Chandramani **Singh**, MD [5], Sagar Vivek **Redkar**, MD [6], Chandra Sekhar **Gillurkar**, MD [7], Jitendra Singh **Kushwaha,** MD [8], Satyajit **Mohapatra**, MD [9], Amit **Bhate**, MD [10], Sanjay **Kumar Rai**, MD [11], Raches **Ella**, MBBS, MS [12], Priya **Abraham**, PhD [2], Sai **Prasad**, MBA[1] , Krishna **Ella**, PhD [1]

[1]Bharat Biotech International Limited, Hyderabad, India

[2]Indian Council of Medical Research-National Institute of Virology, Pune, India

[3] Nizam's Institute of Medical Sciences, Hyderabad, India

[4] Pandit Bhagwat Dayal Sharma Post Graduate Institute of Medical Sciences, Rohtak, India

[5] All India Institute of Medical Sciences, Patna, India

[6] Redkar Hospital, Dargalim, India

[7] Gillurkar Hospital, Nagpur, India

[8] Prakhar Hospital, Kanpur, India

[9] SRM Hospital and Research Centre, Kattankulathur, India

[10] Jeevan Rekha Hospital, Belgaum, India

[11]All India Institute of Medical Sciences, New Delhi, India

[12] Independent Clinical Development Consultant, Cambridge, USA

***Corresponding author:** Dr. Krishna Mohan Vadrevu

Bharat Biotech, Genome Valley, Hyderabad, India – 500 078

Email: kmohan@bharatbiotech.com | Mobile: +91 984 8424500

Registered with the Clinical Trials Registry (India) No. CTRI/2021/04/032942, dated 19/04/2021 and on Clinicaltrials.gov: NCT04471519

Table of Contents

Supplementary data

Table S1 **:** List of clinical sites and Ethical Committees registrations……......……... 04

Figure S1 **:** Decay of neutralising antibody titers ………………………………….... 05

Figure S2 **:** Gender wise PRNT neutralising antibody titers and seroconversion......... 06

Figure S3 **:** PRNT neutralising antibody titers against Variants of Concern……….... 07

Figure S4 **:** Immunoglobulin Subclass Analysis …….……………………………..... 08

Figure S5 **:** IFNγ (T cell) responses …………………..………………………….... 09

Table S2 **:** Summary of all solicited adverse events………………………............ 10

Figure S6 **:** COVID-19 progression in India *vs*,. Neutralising antibodies………....... 11

This supplementary material has been provided by the authors to give readers additional information about their work.

Table S1: List of clinical sites and the respective Ethical Committee registration details, from which the clinical trial protocol got approved.

S. No.	Site Name	Ethics Committee Name	Ethics Committee details
1.	Pandit Bhagwat Dayal Sharma Post Graduate Institute of Medical Sciences (PGIMS), Rohtak Haryana	Institutional Ethics Committee, Pt.B.D. Sharma PGIMS/UHS	ECR/293/Inst/HR/2013/RR-19
2.	All India Institute of Medical Sciences (AIIMS), New Delhi	Institute Ethics Committee All India institute of medical sciences, New Delhi	ECR/547/INST/DL/2014/RR-17
3.	Jeevan Rekha Hospital, Belgaum	Institutional Ethics Committee of Jeevan Rekha Hospital	ECR/1242/INST/KA/2019
4.	Gillukar Multispeciality Hospital, Nagpur	Gillurkar Hospital Ethics Committee	ECR/1374/INST/MH/2020
5.	All India Institute of Medical Sciences (AIIMS), Patna	Institutional Ethics Committee, All India institute of medical sciences, Patna	ECR/1387/INST/BR/2020
6.	SRM Hospital & Research Center, Kattankulathur, Tamilnadu	SRM Medical College Hospital & Research center, Institutional Ethics Committee	ECR/431/INST/TL/2013/RR-19
7.	Nizam’s Institute of Medical Sciences (NIMS) Hospital, Hyderabad, Telangana	NIMS Institutional Ethics Committee	ECR/303/INST/AP/2013/RR-19
8.	Prakhar Hospital, Kanpur	Ethics Committee of the Prakhar Hospital	ECR/1017/INST/UP/2017
9.	Redkar Hospital, GOA	Redkar Hospital and Research Centre Institutional ethics committee	ECR/902/INST/GA/2018

Figure S1: Decay of neutralising antibody titers (shown as GMTs) up to the time of booster assessment following two doses of BBV152 administered on Days 0 and 28.

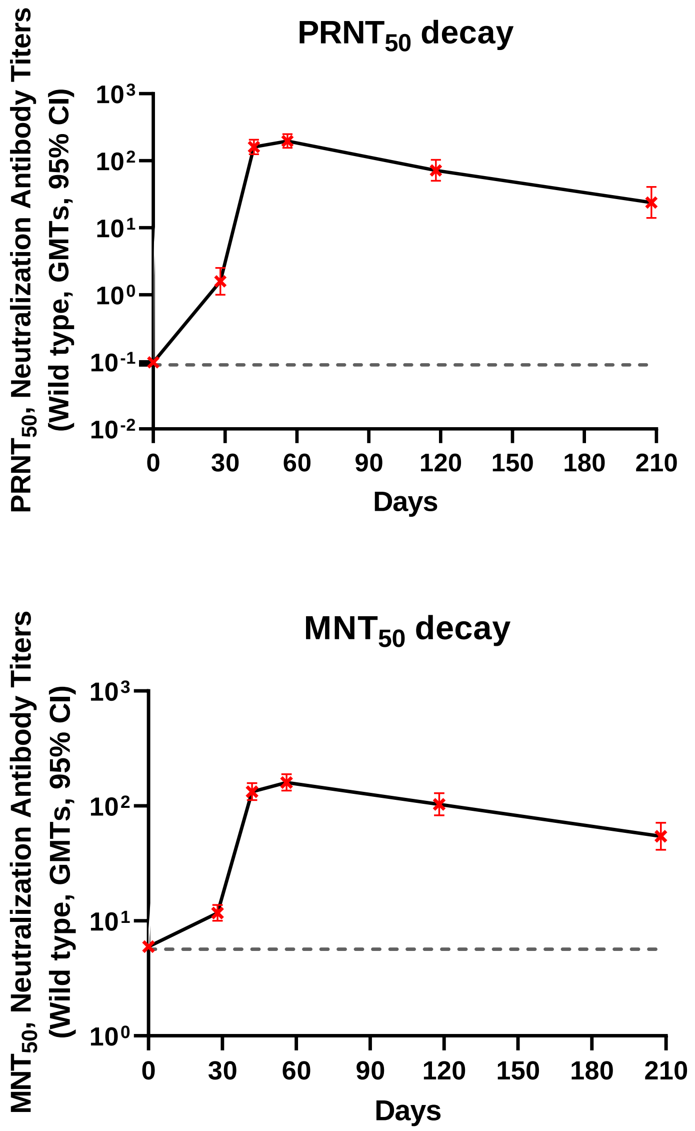

Figure S2: Gender wise $PRNT_{50}$ neutralising antibody titers (shown as individual titers and GMTs) against ancestral (D614G) strain (A). Seroconversion rates defined as a post-vaccination titer that were at least four-fold higher than the baseline titer (B). Samples collected on Day 243 (28days, post third dose) from booster and non-booster individuals. Symbols show GMTs (A) or percent seroconversion (B) and error bars indicate 95% CI.

A. Gender wise $PRNT_{50}$ neutralization antibody titers in booster and non-booster individuals, on Day 243 (Post 3rd Dose)

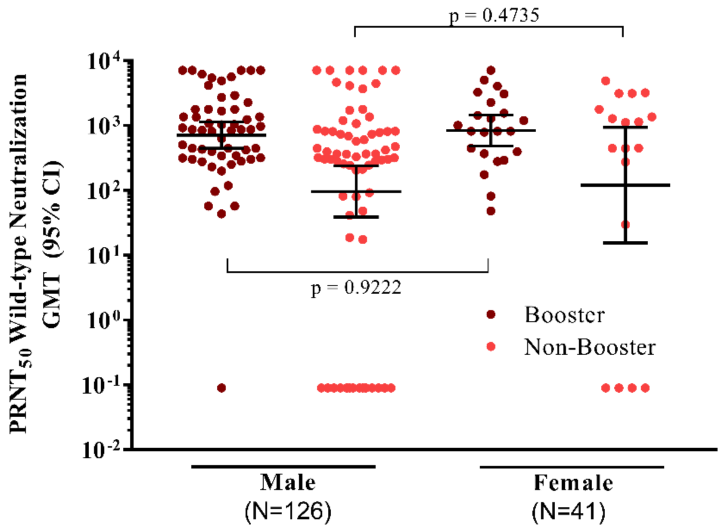

B. Gender wise Seroconversion rates in booster and non-booster individuals, on Day 243 (Post 3rd Dose)

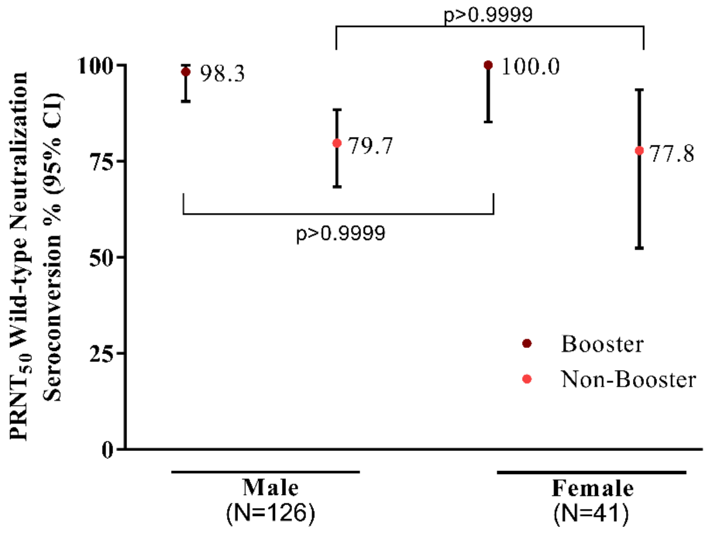

Figure S3: PRNT neutralising antibody titers (GMTs) against the indicated D614G and Variants of Concern six months after third dose of BBV152 administered on Days 215. Samples collected and analysed 6months after the booster dose (Day 395). Symbols show GMTs and error bars indicate 95% CI.

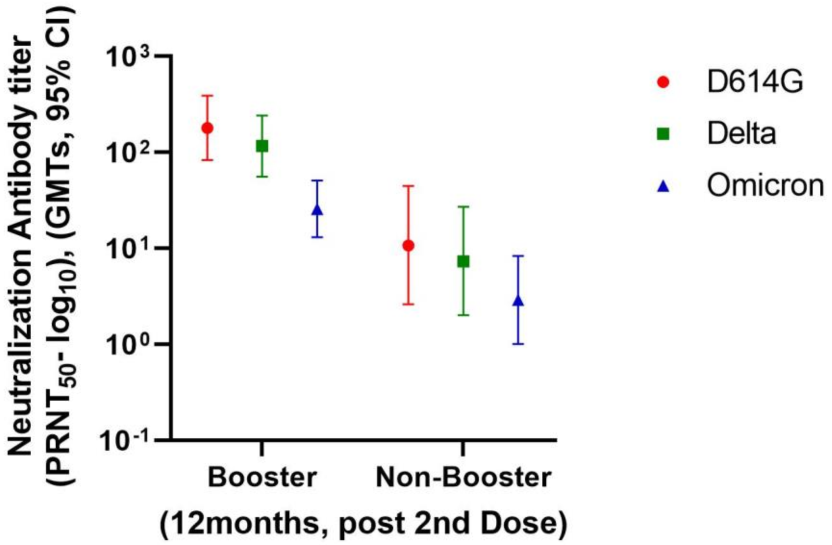

Figure S4: Immunoglobulin Subclass Analysis

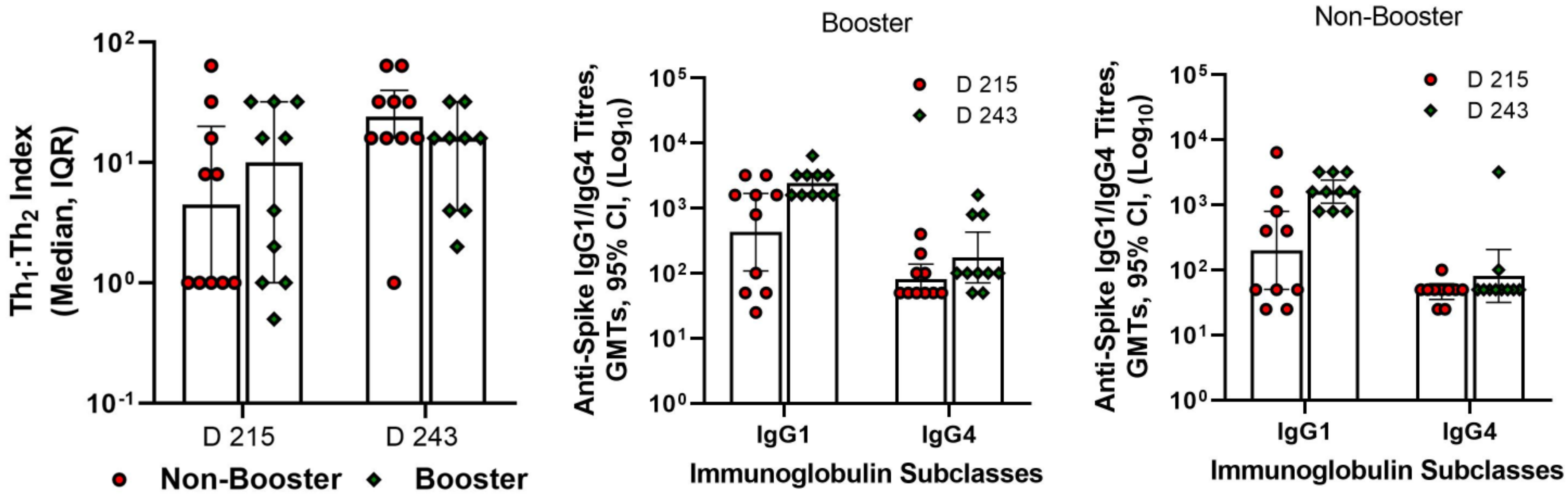

The Th1: Th2 index (medians with IQR) was generated by the ratio of Th1-dependent IgG1 endpoint antibody titer vs Th2-dependent IgG4 endpoint antibody titer, with individual data points shown.

Serum samples were collected on Days 215 and 243, before and after the third dose (Booster or Non-Booster) and IgG1 and IgG4 antibody titers measured by ELISA.

Cut off (Mean + 3 SD) was determined by calculating the absorbance obtained at all serum dilutions (except the lower dilution, 1:50) of known negative control (unvaccinated sera).

Figure S5: IFNγ (T cell) responses, 6 months after dose 2 (Day 208).

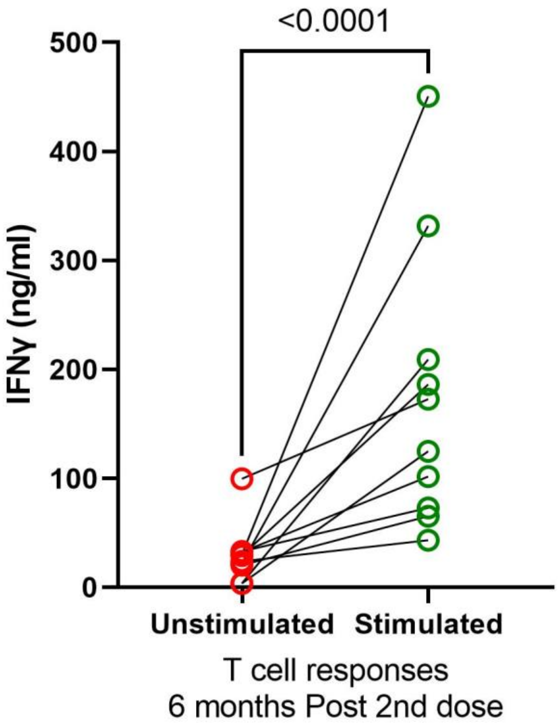

IFNγ levels were estimated in the whole blood assay using whole blood collected from vaccinated subjects on Day 208 (6 months post dose 2). Each open circle represents data from an individual participant and with or without COVID-19 antigen stimulation; difference between unstimulated and stimulated was $P < 0.0001$.

Table S2: Summary of all solicited adverse events post-vaccination for individual treatment groups in safety population

**Parameters**	**Booster** (N = 91)	**Non-Booster** (N = 93)
	**[Events] n participants (%)**	**[Events] n participants (%)**
**Overall**	**[8] 8 (8·7%)**	**[5] 5 (5·3%)**
**Local at Injection Site**	**[8] 8 (8·7%)**	**[2] 2 (2·1%)**
Pain	[5] 5 (5·4%)	[2] 2 (2·1%)
Itching	[2] 2 (2·1%)	-
Redness	[1] 1 (1·0%)	-
**Systemic**	**0**	**[3] 3 (3·2%)**
Fever	0	[2] 2 (2·1%)
Headache	0	[1] 1 (1·0%)

N = Total number of subjects in group; n = Number of participants with Adverse Event in group

Figure S6: COVID-19 progression in India and correlation with neutralising antibody titers

A: $PRNT_{50}$ Titers B: MNT_{50} Titers

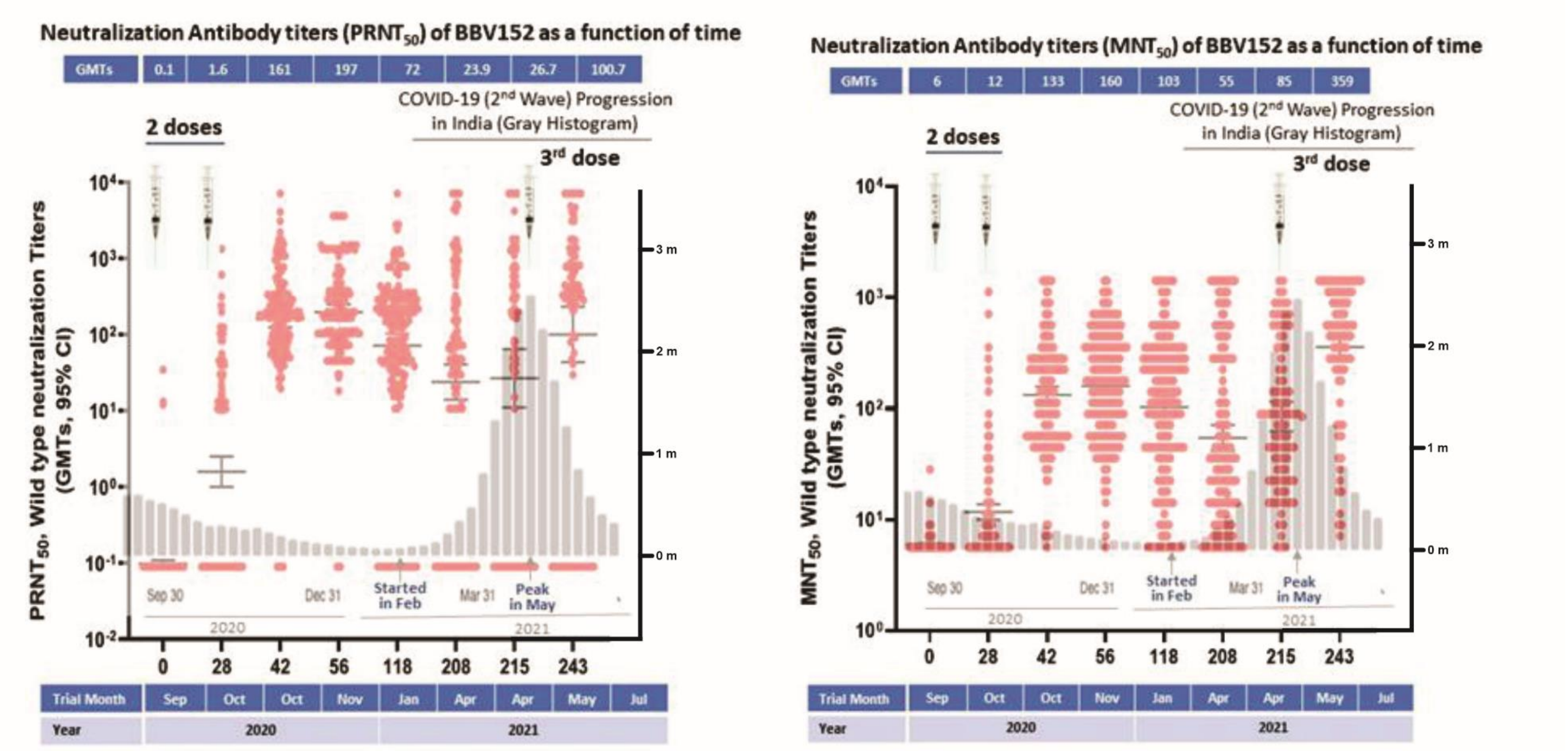

Neutralising antibody titers measured by A. PRNT and B. MNT in unboosted individuals plotted as a function of time after the primary vaccination series in both panels and with COVID-19 progression in India overlaid. Red circles shows the individual antibody titeres, lines the GMTs and 95% CI, and grey columns indicates the surge of COVID-19 cases when the clinical trial was ongoing (scale not shown). Blue row on top indicates GMT at each time point and the blue row at the bottom the corresponding month and year of clinical trial schedule respectively.
