# Supplementary material for: Persistence of immunity and impact of third dose of inactivated COVID-19 vaccine against emerging variants
Source: Sci Rep. 2022 Jul 14;12:12038. doi: 10.1038/s41598-022-16097-3 (PMC9281359; doi:10.1038/s41598-022-16097-3)
Supplement: Supplementary file 2 — Supplementary Information 2. [file 41598_2022_16097_MOESM2_ESM.pdf]

S. No.	Site Name	EC Name	EC details
1.	PGIMS, Rohtak, Haryana	Institutional Ethics Committee, Pt. B.D. Sharma PGIMS/UHS	ECR/293/Inst/HR/2013/RR-19
2.	AIIMS, New Delhi	Institute Ethics Committee<br>All India institute of medical sciences, New Delhi	ECR/547/INST/DL/2014/RR-20
3.	Jeevan Rekha Hospital, Belgaum	Institutional Ethics Committee of Jeevan Rekha Hospital	ECR/1242/INST/KA/2019
4.	Gillukar Multispecialty Hospital, Nagpur	Gillurkar Hospital Ethics Committee	ECR/1374/INST/MH/2020
5.	AIIMS, Patna	Institutional Ethics Committee,<br>All India institute of medical sciences, Patna	ECR/1387/INST/BR/2020
6.	SRM Hospital & Research center, Tamilnadu	SRM Medical College Hospital & Research center,<br>Institutional Ethics Committee	ECR/431/INST/TL/2013/RR-19
7.	NIMS Hospital, Hyderabad, Telangana	NIMS Institutional Ethics Committee	ECR/303/INST/AP/2013/RR-19
8.	Prakhar Hospital, Kanpur	Ethics Committee of the Prakhar Hospital	ECR/1017/INST/UP/2017/RR21
9.	Redkar Hospital, GOA	Redkar Hospital and Research Centre Institutional ethics committee	ECR/902/INST/GA/2018/RR21
